# Basal osteotomy of the first metacarpal using patient-specific guides and instrumentation: biomechanical and 3D CT-based analysis

**DOI:** 10.1007/s00402-023-05122-3

**Published:** 2023-11-24

**Authors:** Cléa Nüesch, Andreas Schweizer, Andreas Weber, Lisa Reissner

**Affiliations:** Division of Hand Surgery, Balgrist University Hospital Zurich, Forchstrasse 340, 8008 Zurich, Switzerland

**Keywords:** Trapeziometacarpal osteoarthritis, Extension osteotomy, Three-dimensional patient-specific planning and instrumentation, Peak load zone, Joint, Articular surface

## Abstract

**Introduction:**

The aim of this study was to investigate the radiological outcomes of proximal closing metacarpal extension osteotomies using patient-specific guides and instruments (PSI) in early-stage trapeziometacarpal osteoarthritis to gain further insight into the joint loading surface and the benefits of the procedure.

**Methods:**

In a prospective observational study, nine patients were included between 11/2020 and 12/2021, undergoing a total of ten proximal metacarpal extension osteotomies for basal thumb osteoarthritis. Computer-assisted surgical planning was performed using computed tomography (CT) and three-dimensional (3D) segmentation, allowing the fabrication of 3D-printed PSIs for surgical treatment. Inclusion criteria were a 1-year follow-up by CT to assess postoperative correction of the positional shift of the first metacarpal (MC1) and the location of peak loads compared with the preoperative situation.

**Results:**

Radiographic analysis of the peak loading zone revealed a mean displacement on the articular surface of the trapezius of 0.4 mm ± 1.4 mm to radial and 0.1 mm ± 1.2 mm to palmar, and on the articular surface of the MC1 of 0.4 mm ± 1.4 mm to radial and 0.1 mm ± 1.2 mm to dorsal.

**Conclusion:**

There were trends indicating that a flatter pressure distribution and a dorsal shift of the peak loading zone may contribute to an improvement in subjective pain and patient satisfaction associated with this surgical procedure. The non-significant radiological results and the minor dorsal-radial shifts in our small study group limit a firm conclusion.

**Level of evidence:**

III.

## Introduction

Trapeziometacarpal osteoarthritis (TMC OA) is worldwide a very common degenerative disease affecting cartilage and bone associated with pain, weakness, loss of motion, adduction deformity and limitations in activities of daily living [1–4].

For many patients in the early stages, according to Eaton and Littler, conservative treatment may already be sufficient [1]. For more severe osteoarthritis, however, elaborate treatment strategies are required. A variety of surgical procedures have been developed for this purpose. In the early stages, ligament reconstruction, extension osteotomy at the base of the first metacarpal (MC1) bone or denervation are considered the most effective methods. In advanced stages with increased cartilage damage, mobility-preserving procedures such as resection arthroplasty and prosthetic treatment are used in addition to arthrodesis [5–8].

Since the work of Wilson, the dorsal extending osteotomy of the MC1 has only been described in small patient studies, which have shown good patient satisfaction with a high grip strength [2, 4, 9–12]. In this procedure, a closed wedge osteotomy is performed at the MC1 base with a dorsal closure of 20–30°, which alters the pressure loading pattern to reduce the subluxation of the thumb saddle joint and thus increase the stability of the joint [13–15]. This surgical method showed good results in both mild and moderate stages. However, at an advanced stage, no convincing effects have yet been shown [14].

Despite the promising outcome data, there has been no study analysing the osteotomy performed and its effects on TMC joint unloading using computed tomography (CT) [2, 9, 11, 12]. Three-dimensional (3D) CT and the resulting generated 3D bone models allow a more precise evaluation of the deformity in 3D space, which can be crucial for the preoperative planning and execution of a precise reduction osteotomy. New innovations in 3D printing technology have facilitated the production of patient-specific guides that enable the transfer of computer-assisted planning to the intraoperative field. Regarding TMC OA, only one study analysed the surgical outcome in terms of the precision of the corrective osteotomy, performed with PSI, using postoperative CT data [10]. In their retrospective study, a 20° extending and 5° ulnar adducting osteotomy was performed after a 3D analysis of the TMC joint to achieve an optimal pinch-grip position and articular surface contact. The results were very satisfactory.

Therefore, this prospective study aimed to analyse the biomechanics of ten cases of early-stage trapeziometacarpal osteoarthritis cases after extending and ulnar adducting osteotomy of the MC1 and to compare the joint loading area prior surgery to 1 year after surgery. For this purpose, preoperative and postoperative CT images were compared with 3D models in static, unloaded key pinch position to assess the difference in the peak load zone. The hypothesis was that the main joint contact would shift dorsally so that the joint surfaces would come into closer congruent contact, flattening the pressure distribution and relieving the load pattern on the joint surfaces.

## Methods

Between November 2020 and December 2021, we included nine patients with early TMC OA in a prospective observational study who underwent Wilson extension osteotomy using preoperative 3D planning and PSI at our institution. A total of ten osteotomies were considered. Inclusion criteria were early-stage TMC OA according to Eaton stages I-II, a follow-up of at least 1 year with CT scan follow-up, a signed informed consent form and a minimum age of 18 years.

Ethical approval was given by the responsible ethics committee and consent was obtained from all patients for the use of patient data.

**Fig. 1:**
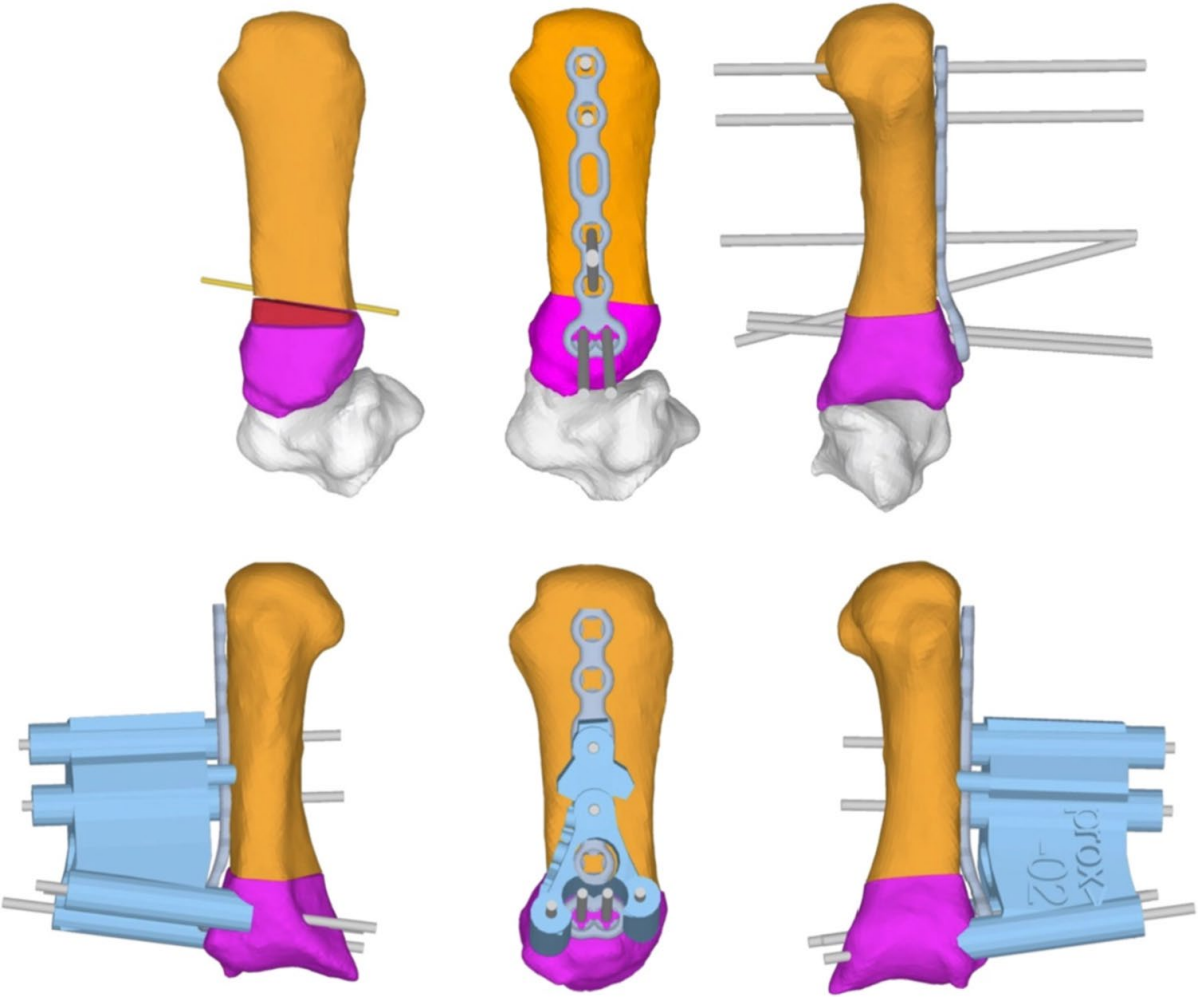
Surgical analysis and plan with the osteotomy templates. Depicted is an osteotomy wedge (red) with 20° dorsalextending and 5° ulnar adducting osteotomy. To perform the planned osteotomy and to position the Kirschner wires, the patient-specific guides and instruments (blue) were used [10]

For preoperative planning, a static CT scan of each affected hand was obtained in unloaded key pinch position in 1-mm-thick scan slices (Siemens Somotom Edge Plus, Germany), which after segmentation with Mimics software (MIMICS version 23, Leuven, Belgium) was used to create 3D surface models. The planning software CASPA (CASPA; Balgrist CARD AG, Zurich, Switzerland) was used to simulate the osteotomy and create the PSI templates, which were used as pre-drill templates and guide templates for the osteotomy incision (Fig. 1). A previously published study at the same institution, which applied the same procedures and was able to corroborate their precision, explains the more precise approach to creating these PSIs and outlines the same surgical procedure [10].

### Radiographic analysis

A standardised radiographic examination was performed on the patients preoperatively and at least 1 year after surgery.

**Fig. 2:**
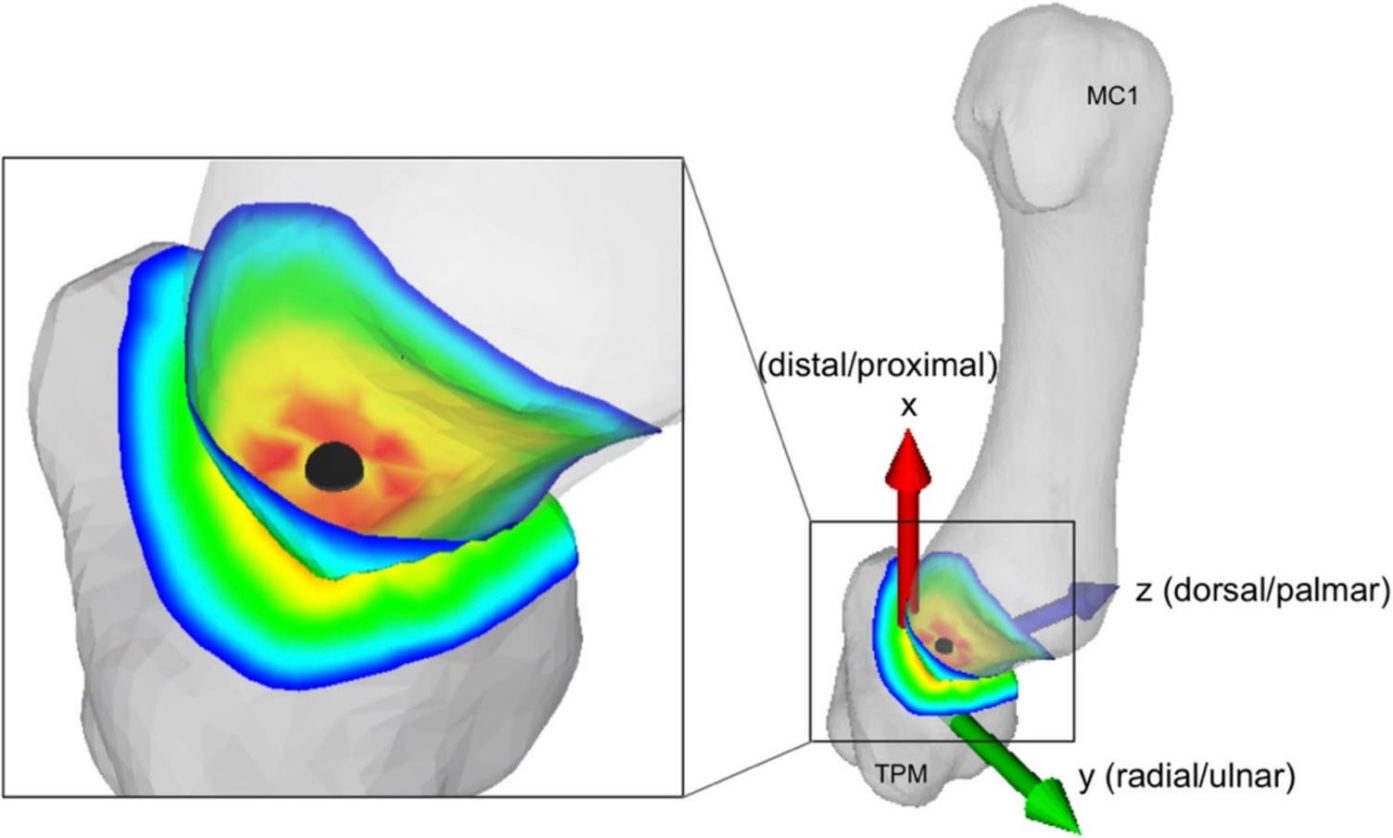
A 3D model showing an unloaded first metacarpal (MC1), a fixed trapezium (TPM) and an anatomical coordinate system aligned with the shaft of the MC1 positioned in the centre of the trapezium, with the x-axis corresponding to the distal–proximal axis, the y-axis corresponding to the radial–ulnar axis and the z-axis corresponding to the dorsal-palmar axis. The black point refers to the centroid of the peak load zone

Postoperative CT with the corresponding 3D models was used for comparison with the preoperative plan. In each case, the 3D surface models of the trapezium with the corresponding MC1 bone were used to determine the changes in the TMC joint. The trapezium was chosen as the reference point, which is fixed in space and towards which the metacarpal bone shifts. Using the Iterative Closest Point (ICP) surface registration method, the preoperative trapezium in static, unloaded key pinch position was registered and aligned with the postoperative trapezium in identical position. An anatomical coordinate system aligned with the shaft of the MC1 was set in the centre of the trapezium with exactly the same orientation as in the preoperative planning, with the x-axis corresponding to the distal–proximal axis, the y-axis corresponding to the radial–ulnar axis and the z-axis corresponding to the dorsal–palmar axis (Fig. 2). Subsequently, the difference between the preoperative and postoperative CT was measured by calculating the translation displacement of the metacarpal bone fragment proximal to the osteotomy with respect to the center of the trapezium with ICP. Between the two positions of the basal metacarpal fragment, the displacement was expressed by three translation components along the previously established coordinate system.

For the measurement of the minimum joint space between the trapezium and the MC1, the bone-to-bone distance maps were calculated with the CASPA software using a combined distance field and mesh surface representation of the bone models for each of the scanned positions and were displayed using a customized colour scale, which was used as a surrogate for intra-articular loading. The minimum joint space was defined as the minimum distance between the two closest points of the opposing bones.

**Fig. 3:**
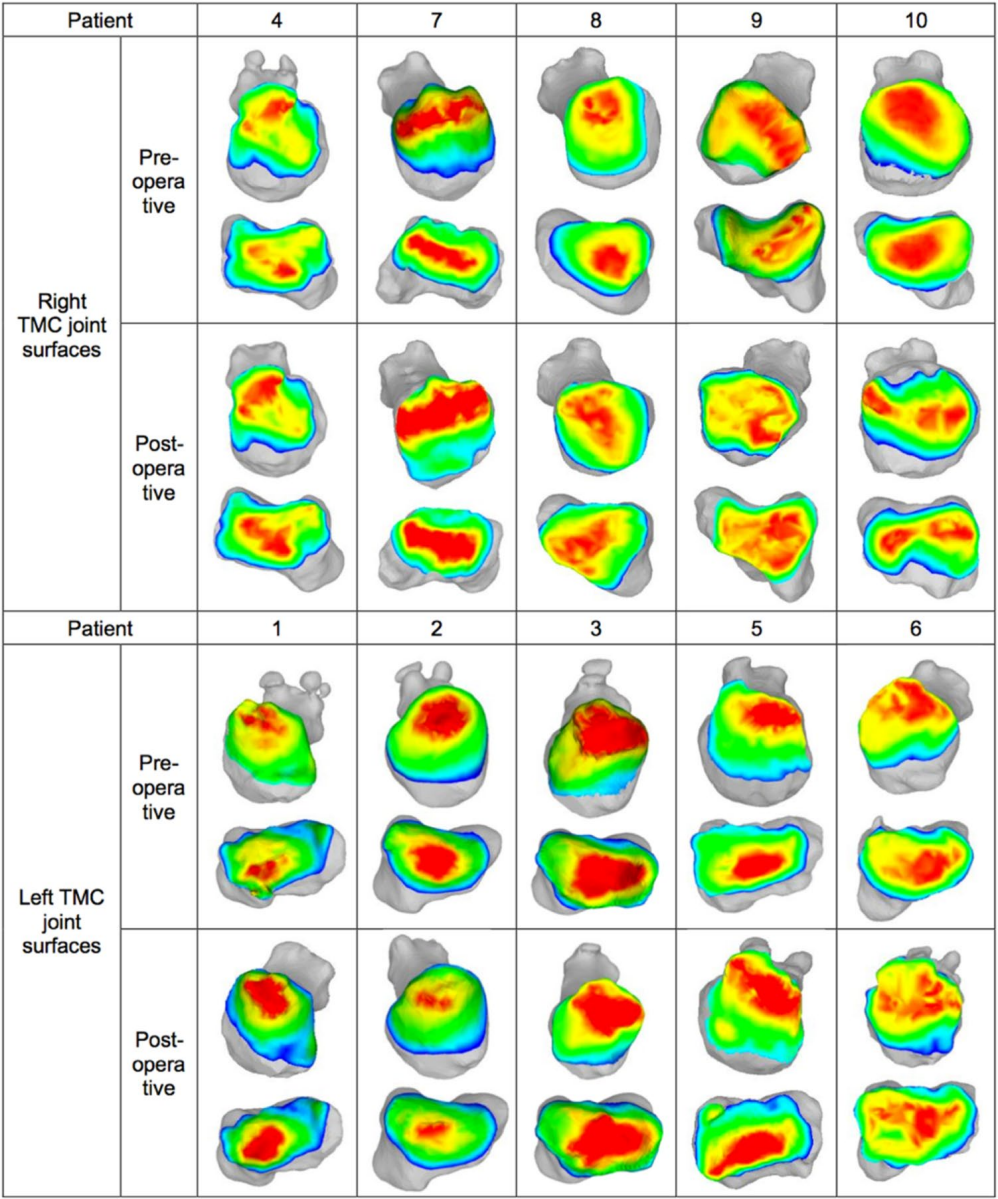
3D bone models of the ten patients representing the loading pattern observed on the articular surface of each bone, with colour intensity indicating the bone distance between the first metacarpal (MC1) and the trapezium (TPM). The joint space is defined as the area where the bone-to-bone distance is less than or equal to 1.5 mm (yellow), while the peak load zone is defined as a distance of 0.5 mm (red)

To define the articular surface from the respective bone models, a normal physiological joint space of 1.5 mm was assumed, based on the combined thicknesses of the healthy MC1 and trapezoidal cartilage layers according to Koff et al. [16]. Using the CASPA program, the articular surface of a bone model was selected applying the clip chart function based on the distance field measurements of the opposite bone model, which included all points within the threshold of 1.5 mm. In order to define the peak load zone more precisely, the same procedure was used to define a close-up range of 0.5 mm, which includes all distance points below this threshold (Fig. 3).

Eventually, the displacement of the peak load zone from preoperative to postoperative was measured. For this purpose, the geometric centre of gravity of the peak load zone, previously determined with a close-up range of 0.5 mm to the opposite bone, was taken. The geometric centre of gravity $s$ was calculated automatically by the CASPA program using the following formula: [17]

$$s=\frac{1}{m}\sum_{i=1}^{m}{x}_{i},$$

where ${x}_{i}$ correspond to the surface points defined by a triangular mesh and $m$ is the number of points. The resulting preoperative and postoperative geometric centres of the peak load zones could then be compared and the displacement expressed in three translational components. For this purpose, the same anatomical coordinate system was used as described earlier.

### Statistical analysis

Descriptive statistics are reported as mean ± standard deviation (range, minimum to maximum). The statistical analysis was carried out with IBM SPSS Statistics 29. The nonparametric Wilcoxon signed-rank test was used for the nonnormally distributed preoperative and postoperative datasets. P-values below 0.05 were considered significant. Correlations were carried out with correlation coefficients according to Spearman.

## Results

During the study period, a total of ten osteotomies were performed in nine patients, with a minimum follow-up of 1 year. They were six women and three men with an average age of 45 years (range 30 to 58, ± 9.0 years) at surgery. According to the preoperative radiographic Eaton classification, two thumbs were classified as stage I and eight thumbs as stage II [18]. The surgery was performed on five right and five left hands, with six of the cases being the dominant hand. Eight patients were able to return postoperatively to the same job with the same level of employment. One patient was able to fully return to work after the operation from the incapacity to work before operation. And one patient was unemployed both before and after the operation.

For the entire study group, the overall median follow-up time for which no revision was required was 1 year. No intra- or postoperative complications were observed.

### Radiological results

**Fig. 4:**
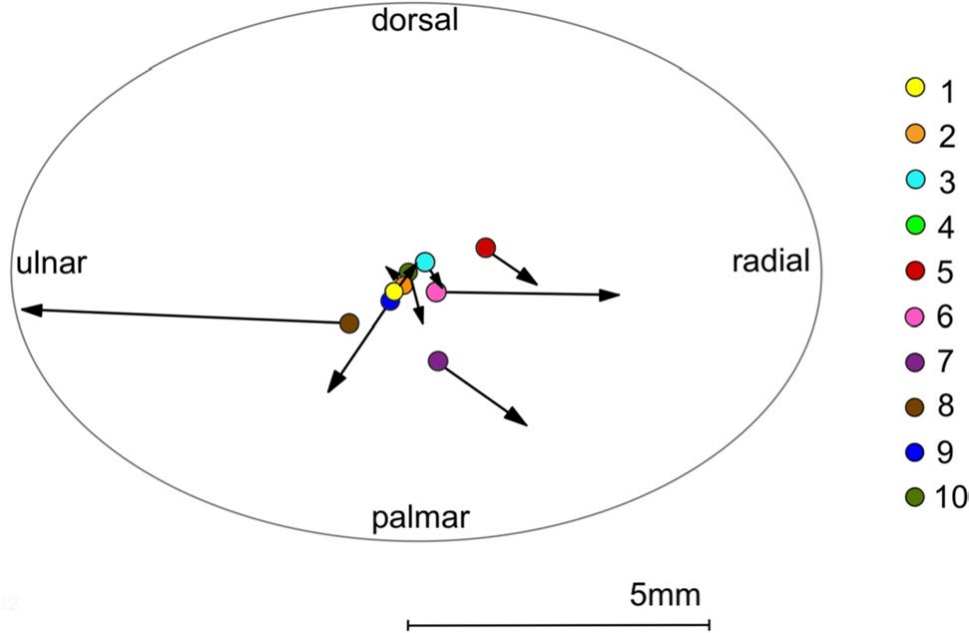
Translation displacement of the base of the first metacarpal from preoperative to postoperative of each individual (coloured points)

The results of the 3D translation displacement of the base of the MC1 based on preoperative and postoperative CT are shown in Table 1 and Fig. 4. The assessment of the displacement is carried out in each case according to the defined coordinate system in relation to the three anatomical planes. The displacement of the base of the MC1 was very heterogeneous and was less than 1 mm in seven out of ten patients.

**Table 1:** Preoperative and postoperative translation (mm) of the basis of the first metacarpal

	Translation (mm)	
Patient	Radial + Ulnar –	Dorsal + Palmar –
1	0.3	0.4
2	– 0.1	0.1
3	0.2	– 0.3
4	0.1	– 0.1
5	0.8	– 0.5
6	3.2	0.0
7	1.5	– 1.0
8	– 5.8	0.2
9	– 1.0	– 1.5
10	0.2	– 0.7

Comparison of the preoperative and postoperative 3D data in the static, unloaded key pinch position revealed a mean displacement of the base of the MC1 of 0.1 mm ± 2.3 mm in ulnar and 0.3 mm ± 0.6 mm in palmar deviation.

The minimum joint space distance remained approximately unchanged from preoperative to postoperative, with a mean distance of 0.1 mm ± 0.2 mm preoperatively to 0.2 mm ± 0.3 mm postoperatively. Because of the expected joint space narrowing in patients with osteoarthritis and the small area that is difficult to capture with CT resolution, joint space measurements were close to zero in each configuration.

Area measurement of the peak load zone within a distance of 0.5 mm from the opposing bone showed an increase in the peak load zone for both the trapezium by 15.0% (*p* = 0.515) and the base of the MC1 by 31.8% (*p* = 0.407) from preoperative to postoperative. The correlation test between the size of the peak load zone and the pain according to the MHQ showed a non-significant negative correlation for the trapezium (*r* = − 0.226; *p* = 0.559), as well as for the MC1 (*r* = − 0.332; *p* = 0.383).

**Fig. 5:**
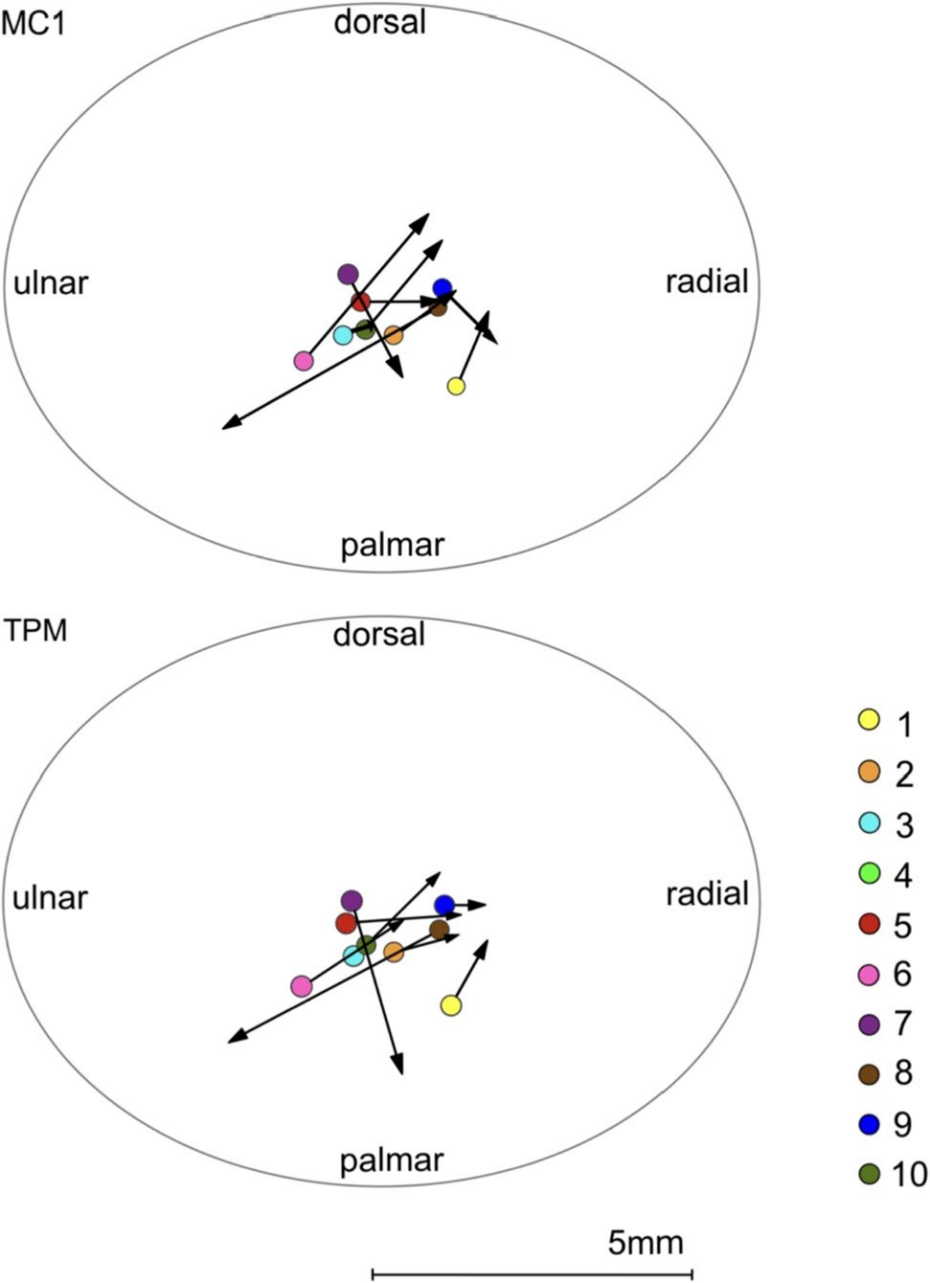
Translation displacement of the peak load zone on the articular surface of the trapezium (TPM) and the first metacarpal (MC1) from preoperative to postoperative of each individual (coloured points)

The results of the displacement of the peak load zone are given as translational displacement according to the defined coordinate system (Table 2; Fig. 5). The peak load zone of the trapezium articular surface shifted postoperatively by an average of 0.4 mm ± 1.4 mm (*p* = 0.110) to radial and 0.1 mm ± 1.2 mm (*p* = 0.515) to palmar. The joint surface of MC1 showed a mean translational displacement of 0.4 mm ± 1.4 mm (*p* = 0.110) to radial and 0.1 mm ± 1.2 mm (*p* = 0.594) to dorsal. The measurement of the peak load zone of the trapezium and MC1 could not be evaluated in one patient, because there was a joint gap larger than 0.5 mm for the measurement and thus a comparison with the other patients was not possible.

**Table 2:** Preoperative and postoperative translation displacement (mm) of the peak load zone

	Translation (mm) – TPM		Translation (mm) – MC1	
Patient	Radial + Ulnar –	Dorsal + Palmar –	Radial + Ulnar –	Dorsal + Palmar –
1	0.5	0.9	0.4	1.0
2	0.8	0.2	0.8	0.6
3	0.0	0.0	0.3	0.1
4	–	–	–	–
5	1.6	0.1	0.9	0.0
6	1.4	0.9	1.8	2.1
7	0.7	– 2.4	0.8	– 1.4
8	– 3.1	– 1.6	– 3.1	– 1.7
9	0.4	0.0	0.7	– 0.7
10	1.0	1.0	1.1	1.3

*TPZ* Trapezium, MC1 = First Metacarpal

## Discussion

The aim of this study was to take advantage of preoperative 3D planning in combination with PSI to achieve high precision of extension osteotomy and to assess radiological outcomes using a CT and their analysis in 3D imaging. We suspect that a precise osteotomy targeting 20° of extension and 5° of ulnar adduction to achieve optimal contact of the articular surfaces in the pinch-grip position may lead to an explanation of the improvement of patient symptoms from a biomechanical point of view in a 3D model analysis.

First described by Wilson in 1973, osteotomy of the basal metacarpal bone for TMC-OA was able to relieve joint pressure in the early stages of osteoarthritis and showed satisfactory results in several studies in short- to medium-term follow-up [8, 9, 13, 15, 19]. The biomechanical study by Pellegrini et al. demonstrated the efficacy of extension osteotomy, with unloading of the palmar contact area to the normal dorsal compartment in non-arthritic and moderately arthritic thumbs [14]. Many researchers agree that pathological joint loads and peak loads in the joint are important causes for the progression of osteoarthritis [19, 20], but so far, the positive effect of extension osteotomy could not be explained from a biomechanical point of view using radiological analysis. In recent studies, the increasingly recognised advantages of three-dimensional preoperative planning in combination with PSI have been used to achieve more precise results in corrective osteotomy, anatomical reduction in scaphoid reconstruction or correction of metacarpal deformities than with the previously used freehand techniques [21–23].

We evaluated radiographic outcomes after extension osteotomy of the MC1 in TMC OA and created 3D representations of distance fields using a CT-based method to evaluate variations in the peak load zones of the joint space in vivo. Based on a previously conducted study verifying the precision of osteotomy using PSI [10], our results of radiological analysis of the displacement of the peak loading zone on the articular surfaces did not allow a conclusive explanation for our assumption of a dorsal–radial displacement after osteotomy due to the heterogeneous and small values below 1 mm in the majority of patients. Nevertheless, interesting trends emerged that could contribute to a flatter pressure distribution and a dorsal shift of the peak stress zone. There is potential for further studies addressing these findings.

3D analysis of the base of the MC1 bone fragment showed a palmar displacement tendency from preoperative to postoperative, which could have a favourable effect on the progression of OA, as dorsal subluxation of MC1 is observed in advanced TMC-OA. In terms of joint loading, the palmar displacement of the basal MC1 bone fragment causes a shift in the center of loading from palmar to dorsal as the palmar tip of the MC1 is unloaded [4, 12, 24, 25]. This is due to the compressive forces acting on the MC1 tend to rotate the trapezium in the dorsal–ulnar direction, with these joint forces displacing the MC1 in the dorsal–ulnar direction, leading to erosion of the ulnar–palmar joint surfaces [11]. Halilaj et al. and Ateshian et al. also confirmed that the peak load zones in the neutral thumb position were mainly in the palmar–central and central–radial directions [26, 27]. This consideration was verified by evaluating the displacement of the peak load zone of the articular surfaces, with the MC1 articular surface showing stronger tendencies of a dorsal–radial displacement, with a mean value of 0.4 mm to radial and of 0.1 mm to dorsal. The trapezium showed an overall shift of 0.1 mm to palmar, although seven patients showed a shift to dorsal. This is attributed to two patients who had inexplicably larger displacements to palmar, reversing the average value as these are very small values. This hypothesis of a translational shift of the peak load zone is not confirmed, but there is nevertheless a trend in our data for a dorsal–radial shift of the peak load zone with flatter pressure distributions, which can be substantiated by the results of Pellegrini et al. [14]. These authors were able to confirm, in contrast to our in vivo CT-based analysis method, that improved biomechanical efficacy after performed osteotomy was shown in all joints except the most advanced osteoarthritic joints [14]. Interestingly, the study by Koff et al. on the sequential wear pattern of the thumb cartilage in arthritis in the TMC joint was able to show that the degradation of the cartilage surface begins in the dorsal–radial quadrants of the articular surface and quickly moves to the palmar quadrants in stages II, III and IV [16].

Furthermore, in this study, we tried to determine a statement about the minimum joint gap and the area dimension of the peak load. One limitation was the measurement of small joint space distance, due to conversion of CT scans to 1-mm slice images, which, nevertheless, tended to show slightly larger joint spaces in two patients with stage I and two patients with stage II, which increased slightly postoperatively. A biomechanical study by D'Agostino et al. was able to show in a comparison between healthy patients and patients with TMC OA that the minimum joint space was significantly smaller in the OA group compared to healthy subjects [28]. These observations are also in agreement with Halilaj et al., who confirmed the occurrence of joint space narrowing in late-stage OA patients [26].

The surface area of the peak loading zone showed an increase in surface area after extension osteotomy, which can be due to a more congruent articular surface disposition, resulting in a more homogeneous distribution of load on the joint surface. The correlation test, which showed a negative correlation between the area of the peak load zone of and pain, suggests that the area of the peak load zone increases as pain decreases.

The main limitation of this study is the small number of patients. Larger multicentre studies would be needed to assess the displacement of the base of the MC1 and the peak load zones in the TMC joint using computed tomography. A further problem could be the spatial CT resolution, which was set to 1 mm increments in this study. Higher CT resolution might reveal details that would be important to show the advantage of the extension osteotomy in terms of loading patterns on the articular surfaces. The analgesic, pain-relieving effect could also be attributed to the additional denervation than to the actual corrective osteotomy. This would be an interesting point to investigate in a further study.

In this study, CT-based analysis showed trends of palmar displacement of the base of the MC1 associated with displacement of the peak loading zone in the dorsal–radial direction. We thus assume that a 3D patient-specific planning and instrumentation performed osteotomy of the MC1 achieves a force allocation to a mean vertical load in order to reduce the shear stress on the cartilage. The study has with its size not the ambition to form conclusive guidelines. It is intended to stimulate the reader to a broader clinical understanding through new analytical approaches.

## References

[1] Eaton RG, Littler JW (1973). Ligament reconstruction for the painful thumb carpometacarpal joint. J Bone Joint Surg Am.

[2] Gwynne-Jones DP, Penny ID, Sewell SA, Hughes TH (2006). Basal thumb metacarpal osteotomy for trapeziometacarpal osteoarthritis. J Orthop Surg (Hong Kong).

[3] Pellegrini VD (1991). Osteoarthritis of the trapeziometacarpal joint: the pathophysiology of articular cartilage degeneration. I. Anatomy and pathology of the aging joint. J Hand Surg Am.

[4] Wilson JN, Bossley CJ (1983). Osteotomy in the treatment of osteoarthritis of the first carpometacarpal joint. J Bone Joint Surg Br.

[5] Gervis WH, Wells T (1973). A review of excision of the trapezium for osteoarthritis of the trapezio-metacarpal joint after twenty-five years. J Bone Joint Surg Br.

[6] Muller GM (1949). Arthrodesis of the trapezio-metacarpal joint for osteoarthritis. J Bone Joint Surg Br.

[7] Swanson AB (1972). Disabling arthritis at the base of the thumb: treatment by resection of the trapezium and flexible (silicone) implant arthroplasty. J Bone Joint Surg Am.

[8] Wilson JN (1973). Basal osteotomy of the first metacarpal in the treatment of arthritis of the carpometacarpal joint of the thumb. Br J Surg.

[9] Chou FH, Irrgang JJ, Goitz RJ (2014). Long-term follow-up of first metacarpal extension osteotomy for early CMC arthritis. Hand (N Y).

[10] Kriechling P, Reissner L, Zindel C, Andronic O, Schweizer A (2022). Precision of the Wilson corrective osteotomy of the first metacarpal base using specific planning and instruments for treatment of basal thumb arthritis. Arch Orthop Trauma Surg.

[11] Parker WL, Linscheid RL, Amadio PC (2008). Long-term outcomes of first metacarpal extension osteotomy in the treatment of carpal-metacarpal osteoarthritis. J Hand Surg Am.

[12] Tomaino MM (2000). Treatment of Eaton stage I trapeziometacarpal disease with thumb metacarpal extension osteotomy. J Hand Surg Am.

[13] Hobby JL, Lyall HA, Meggitt BF (1998). First metacarpal osteotomy for trapeziometacarpal osteoarthritis. J Bone Joint Surg Br.

[14] Pellegrini VD, Parentis M, Judkins A, Olmstead J, Olcott C (1996). Extension metacarpal osteotomy in the treatment of trapeziometacarpal osteoarthritis: a biomechanical study. J Hand Surg Am.

[15] Shrivastava N, Koff MF, Abbot AE, Mow VC, Rosenwasser MP, Strauch RJ (2003). Simulated extension osteotomy of the thumb metacarpal reduces carpometacarpal joint laxity in lateral pinch. J Hand Surg Am.

[16] Koff MF, Ugwonali OF, Strauch RJ, Rosenwasser MP, Ateshian GA, Mow VC (2003). Sequential wear patterns of the articular cartilage of the thumb carpometacarpal joint in osteoarthritis. J Hand Surg Am.

[17] Protter MH, Morrey CB (1970) College Calculus with Analytic Geometry. In: Addison-Wesley, 2nd edn. Boston

[18] Eaton RG, Glickel SZ (1987). Trapeziometacarpal osteoarthritis. staging as a rationale for treatment. Hand Clin.

[19] Bachoura A, Yakish EJ, Lubahn JD (2018). Survival and long-term outcomes of thumb metacarpal extension osteotomy for symptomatic carpometacarpal laxity and early basal joint arthritis. J Hand Surg Am.

[20] Brandt KD, Dieppe P, Radin E (2009). Etiopathogenesis of osteoarthritis. Med Clin North Am.

[21] Kunz M, Ma B, Rudan JF, Ellis RE, Pichora DR (2013). Image-guided distal radius osteotomy using patient-specific instrument guides. J Hand Surg Am.

[22] Schweizer A, Mauler F, Vlachopoulos L, Nagy L, Fürnstahl P (2016). Computer-assisted 3-dimensional reconstructions of scaphoid fractures and nonunions with and without the use of patient-specific guides: early clinical outcomes and postoperative assessments of reconstruction accuracy. J Hand Surg Am.

[23] Hirsiger S, Schweizer A, Miyake J, Nagy L, Fürnstahl P (2018). Corrective osteotomies of phalangeal and metacarpal malunions using patient-specific guides: CT-based evaluation of the reduction accuracy. Hand (N Y).

[24] Molitor PJ, Emery RJ, Meggitt BF (1991). First metacarpal osteotomy for carpo-metacarpal osteoarthritis. J Hand Surg Br.

[25] Futami T, Nakamura K, Shimajiri I (1992). Osteotomy for trapeziometacarpal arthrosis. 4(1–6) year follow-up of 12 cases. Acta Orthop Scand.

[26] Halilaj E, Moore DC, Laidlaw DH, Got CJ, Weiss AP, Ladd AL, Crisco JJ (2014). The morphology of the thumb carpometacarpal joint does not differ between men and women, but changes with aging and early osteoarthritis. J Biomech.

[27] Ateshian GA, Ark JW, Rosenwasser MP, Pawluk RJ, Soslowsky LJ, Mow VC (1995). Contact areas in the thumb carpometacarpal joint. J Orthop Res.

[28] D'Agostino P, Dourthe B, Kerkhof F, Harry Van Lenthe G, Stockmans F, Vereecke EE (2017). In vivo biomechanical behavior of the trapeziometacarpal joint in healthy and osteoarthritic subjects. Clin Biomech (Bristol, Avon).

